# Evidence of an application of a variable MEMS capacitive sensor for detecting shunt occlusions

**DOI:** 10.1038/srep46039

**Published:** 2017-04-05

**Authors:** David J. Apigo, Philip L. Bartholomew, Thomas Russell, Alokik Kanwal, Reginald C. Farrow, Gordon A. Thomas

**Affiliations:** 1 New Jersey Institute of Technology, Department of Physics, Newark, NJ 07102, USA; 2 New Jersey Institute of Technology, Department of Material Science and Engineering, Newark, NJ 07201, USA

## Abstract

A sensor was tested *subdural* and *in vitro,* simulating a supine infant with a ventricular-peritoneal shunt and controlled occlusions. The variable MEMS capacitive device is able to detect and forecast blockages, similar to early detection procedures in cancer patients. For example, with gradual occlusion development over a year, the method forecasts a danger over one month ahead of blockage. The method also distinguishes between ventricular and peritoneal occlusions. Because the sensor provides quantitative data on the dynamics of the cerebrospinal fluid, it can help test new therapies and work toward understanding hydrocephalus as well as idiopathic normal pressure hydrocephalus. The sensor appears to be a substantial advance in treating brain injuries treated with shunts and has the potential to bring significant impact in a clinical setting.

The goal in previous work^1^,^2^ was to design a sensor that could detect minute pressure ranges and flow rates of fluids such as those associated with cerebrospinal fluid (CSF). By utilizing such a device in-line with current existing shunt technology, it could be possible to reduce brain damage and unnecessary surgery in patients with brain shunts to treat hydrocephalus. Diagnosis of improper flow through a shunt has relied on patient symptoms and advanced medical imaging. In 1956, Spitz and Holter^3^,^4^ invented a shunt and valve system that successfully treated conditions that caused brain damage and death because of accumulated pressure and impaired flow of the CSF (a condition known as hydrocephalus). Hakim improved the shunt significantly with the invention of a variable pressure valve which is set externally to certain pressures^4^. Subsequent work^5^ has produced useful improvements in the valves. Two pervasive problems remain: first that the shunts develop blockages at times, over years, that are difficult to anticipate, and second, that the difficulty in monitoring the pressure and flow of the fluid have held back advancements in understanding the illnesses, including idiopathic normal pressure hydrocephalus^6^,^7^. The results reported here address the problem of how to supplement symptomatic analysis with quantitative measurements of the fluid flow and pressure to assess progression of a patient’s condition.

The current diagnosis of this class of illnesses has been carefully reviewed by Gallia, *et al*.^6^ who state that diagnoses are given by monitoring the symptoms (gait impairment, dementia, and urinary incontinence) of the patient and by advanced imaging. Since the symptoms may be shared with other medical conditions, improved diagnosis is difficult. Commercial shunt valves assist the treatment of hydrocephalus by setting the pressure below which the valve will close and prevent back flow. A typical shunt is composed of a ventricular catheter, the shunt tube, and a peritoneal catheter. An external device fixes the pressure, commonly in a range from 1.5 to 14.7 mmHg^6^. Based on a thorough study, Lutz, *et al*.^8^ state that a device that could determine the CSF flow rate and pressure in a shunt would be a great improvement to current designs, and is highly desirable. These smart shunts would add information about pressure and flow to the standard diagnostic and treatment protocol.

Regarding the causes of shunt blockage, Del Bigio^9^ carried out a careful, systematic review of the biological reactions to implanted shunt devices. His data indicated that a key mechanism of shunt failure is that vascularized pedicles of glial tissue or choroid plexus grow into ventricular catheters, primarily as a mechanical phenomenon. If this gradual growth of an occlusion could be observed before a blockage occurs, evidence of the growth would provide valuable information. Del Bigio also concludes: “Cellular debris or blood can cause dysfunction of valve components. Chronic inflammation, which is nonspecific, might contribute to degradation of the components. Care must be taken to prevent early entry of debris or blood into the shunt system. Ventricular collapse onto the shunt must be avoided. Refinement of manufacturing methods or modification of shunt materials could reduce the susceptibility of shunts to infection and improve longevity of the apparatus.”

In an alternative discussion of the causes of shunt blockage, Kandel^10^ has carefully summarized information indicating that normal cerebrospinal fluid consists of water and smaller ions (i.e. glucose and salts) when the blood-brain barrier is effective. Di Rocco, *et al*.^11^ show evidence that this composition implies the fluid and its solutes are not occluding the shunt, but possibly fragments of bone or brain tissue left over from the surgery to implant the shunt. This finding is in agreement with a previous report by Brydon, *et al*.^12^ indicating that sufficient protein deposition does not occur in shunts to cause blockage. In this case, it would be difficult to forecast shunt blockage if the occlusion occurs suddenly. However, there may be a wide distribution of particle sizes, with the smaller ones being more mobile, and therefore a gradual build-up of occlusions. Nonetheless, it would be valuable to know the hydrodynamics, that is, how the changes in pressure and flow occur.

Standard shunts have demonstrated a substantial improvement in the survivability of patients with excess pressure in their brain compared to no treatment since the 1950’s^13^,^14^. In patients effected by idiopathic normal pressure hydrocephalus, improvement rates up to 96% have been reported^6^. But, occlusions (before or after the valve) are the most common cause of failure of current shunts^6^,^15^. In thorough studies performed by Stone, *et al*.^16^ and Tuli, *et al*.^17^ it was shown that after approximately 24 months, over 50% of shunts need revisions. This requires the patient to undergo a second invasive surgery to remove the old shunt and insert a new one. In some cases, shunts were still viable, but in many, an occlusion had occurred. In a careful study, Simon, *et al*.^18^ demonstrated that these revisions increase the risk of infection.

Some fluid pressure measurements have been made. Arbour^19^ has presented a clear explanation of a current procedure to assess intracranial hypertension: invasive surgery that implants sensors via a catheter which is connected to a transducer that can then read the intracranial pressure (ICP) of the patient. The pressure for a healthy individual ranges between 2 and 18 mmHg^20^,^21^,^22^. If the pressure is not found to be within this range, treatment is necessary. Clark, *et al*.^23^ have shown useful measurements of flow with a piezoresistive pressure transducer which depend on a differential in resistance, but this effective design requires an internal power source and thus the lifetime of the device is limited by the battery. A sensor that operates without any internal power could function for the lifetime of the shunt or of the patient.

These studies indicate that answers to key questions would benefit clinicians and caregivers:

1. Has an occlusion occurred?
2. If not, how great is the risk?
3. If so, is it towards the proximal or distal catheter?
4. After a shunt revision, is there an improvement?
5. If additional treatments become available, are they effective?

We have previously reported^1^,^2^ a capacitive microelectromechanical systems (MEMS) device that monitors milliliter dynamics of fluids, which we believe can be utilized to distinguish between open and occluded shunts. This sensor works on the principle that a change in flow rate or pressure results in a measurable shift in the sensor’s capacitance as the plate spacing between membranes changes. The fluid flow begins in the brain and flows through the shunt tubing until it comes to this sensor, where it exerts a pressure on the first flexible membrane. The fluid continues flowing through the shunt tubing until it comes to the second membrane where it produces another deflection. Flow continues through more tubing for reabsorption in the peritoneum. Different flow rates produce different membrane deflections, and in turn, a different value of the capacitance of the sensor. Figure 1 Demonstrates the flow path and basic operating principle of the sensor. The signal of the device is read wirelessly via inductive coupling, thus requiring no internal power, and this signal can be directly related to pressure and flow rate. Our new result discussed in this paper is evidence that this sensitive device can predict shunt failure due to the gradual growth of an occlusion prior to blockage and its location in regards to the sensor.

## Results

### Occlusion Result

Since occlusions are the major issue with shunts, experiments were run with the sensor positioned prior to the shunt control valve under the assumption the valve was open. Any type of occlusion changes the cross-sectional diameter of the tubing; therefore, to test the effects of occlusions in a calibrated manner *in vitro*, digital calipers were utilized to incrementally pinch the tubing. The result of such pinching is a change in cross-sectional area. This experimental data correlates to the case when gradual occlusions are forming in the shunt tubing over time. As debris accumulates within the shunt, the cross-sectional area changes in a similar way to that when the tube is pinched. Clearly, this method does not explore a sudden occlusion, which is unpredictable. However, in the case of sudden occlusions, a quantifiable signal is achieved.

Figure 2 Shows experimental and control data for a proximal occlusion. In the control, the size of the occlusion is zero (that is, the shunt is fully open) and a series of measurements were made over a time period comparable to the variable cross-section experiment. The fluid flowed at 20 ±1 mL/hr and constant pressure, with cross-sectional area of the tubing constant at 0.5 mm^2^. No significant shift in signal was measurable. For the variable cross-section experiment, the size of the simulated occlusion was increased by altering the cross-sectional area of the tube as described above. In the case of a proximal occlusion, the signal change at the onset of the occlusion is dominated by the flow rate decreasing (i.e. flow-dominated). This is determined by the fact that the change in signal has 3 possible origins:

1. When a proximal occlusion is present, the sensor measures primarily a change in flow. A pressure gradient becomes small in the shunt tube when an occlusion forms at the proximal end and concentrates the increased pressure in the brain. The pressure and flow throughout the tube decreases. The key functional dependence that is expected arises from the increase in flow resistance, *R*. For simple geometries this varies inversely with the average radius of the orifice, *r*, according to a prediction containing the following form^24^:


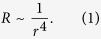


So, this functional form is expected when the reading is dominated by flow.

2. When a distal occlusion is present, the sensor measures primarily a change in pressure, Δ*P*. The pressure increases along the shunt tube, while the flow decreases. The functional form of the reading from the sensor may indicate change, which could be dominated by pressure, and vary with *r*. The predicted form would involve the area of the shunt tube^25^:


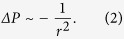


3. A possible hydrostatic pressure caused by any shift in the height of the source. This hydrostatic contribution is held constant in this experiment, where the conditions are equivalent to an implanted shunt with the patient in a constant, supine position. With additional sensors the hydrostatic pressure^25^ can be included:


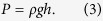


Figure 2 Represents the life of a shunt from the time it is functioning to that when it becomes occluded near the ventricular catheter. When the occlusion is complete, the flow rate is 0 and the sensor provides a direct measurement of the peritoneal pressure. In between, the behavior shows a rapid change from a flat region in which the flow and pressure change very little to one where the signal drops quickly near 0.1 mm^2^, 80% occlusion of the tube area. This behavior is expected for flow-dominated lamellar behavior (see Statistics section).

If the formation of the occlusion proceeds linearly^9^, then it is possible to predict when the occlusion will occur based on the time since shunt implantation. In this case, an 80% point in the process of occlusion allows a prediction of the time when complete blockage will occur. For instance, if the shunt has been implanted for one year, then we can predict that complete blockage will occur in 1/5 of a year, or over 2 months. This substantial knowledge of the likely time of an occlusion may be very helpful to physicians.

**Figure 1:**
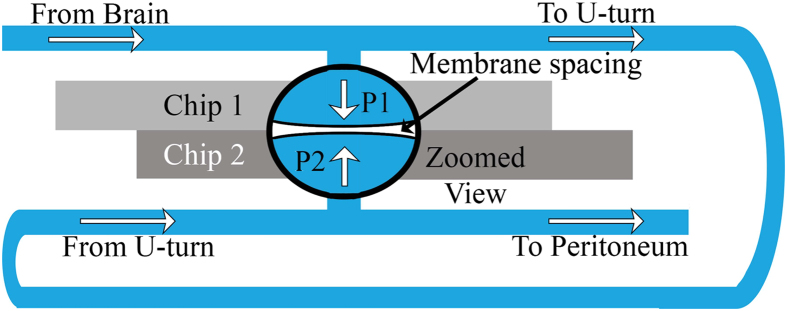
Not-to-scale schematic of fluid flow and operation of the reported MEMS sensor. As fluid flows from the brain through the shunt, it passes the sensor at one point and exerts a pressure that causes the membrane to flex. Flow continues through the shunt tubing and makes contact with the second flexible membrane, exerting a second pressure before flowing out to the peritoneum for reabsorption. The applied pressures produce a change in the capacitance of the device, which in turn produces a measurable shift in the device’s resonant frequency.

Figure 3 Shows the relationship between the pressure-dominated signal and the cross-sectional area of the tube as it decreases from open to fully occluded at a distal point (near the peritoneal cavity). As the cross-sectional area decreases, the signal from the sensor increases. Simultaneously, the flow rate is decreasing. Since the signal trends downward with decreasing flow rate under constant pressure conditions (Fig. 1), the upward trend in signal for a distal occlusion must be due to a pressure change. In fact, when the flow is 0, the sensor provides a direct indication of the ICP. In between, the behavior rises at small changes in area with a statistically significant difference from *P*_0_ at 0.25 mm^2^, 50% occlusion of the tubing area. This behavior is expected for lamellar flow where the pressure dominates (see Statistics). As pressure levels in the brain increase, the trend will continue and not saturate as demonstrated in Fig. 3; however, this data represents the case when the pressure remains constant. For one year since implantation, this behavior allows forecasting of total blockage nearly one year in advance of occurrence. This is 6 times larger than the time for predicting the formation of a proximal occlusion.

## Discussion

Our sensor works in concert with existing pressure control valves and can be positioned before or after said valve. Studies have not yet been performed to determine the optimal location for the sensor in a shunt system. For this discussion, it is assumed that the sensor is placed before the valve. At low pressures, the valve remains closed and no fluid will flow through the shunt, resulting in an increase in pressure if CSF is accumulating in the ventricles. The sensor’s signal will appear to increase as the center deflection of the capacitor membranes decreases. In this configuration, as long as the pressure valve remains closed and the patient remains in a constant supine position, the sensor directly measures the ICP of the individual. However, when the valve opens, this is no longer the case. Once the pressure exceeds the set value of the regulatory valve, fluid flow occurs. At this point, the sensor will function normally. Further study into the dynamics of the sensor with a pressure control valve is necessary. The results presented here represent the condition where the shunt control valve is set to be open at the time of the measurement (to the minimum pressure setting). The result when there is a complete distal occlusion corresponds to the case where the valve is closed (or set to a sufficiently high pressure) and provides a valuable calibration point.

Figure 4 Shows a schematic summary of the method studied here. The method checks a calibration, measures the fluid condition, compares with the previous data and allows an assessment of the need for further tests.

**Figure 2:**
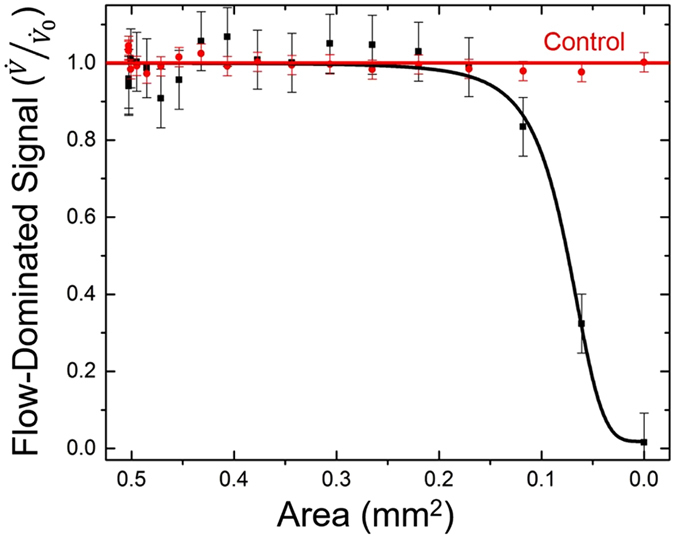
Proximal occlusion results. Data showing flow-dominated signal as a function of area for a simulated proximal occlusion. As the cross-sectional area decreases, it is possible to detect when an occlusion is forming at the proximal catheter in the shunt system. Once the tubing is completely blocked, the signal saturates at 0 mL/hr as the sensor feels no change.

**Figure 3:**
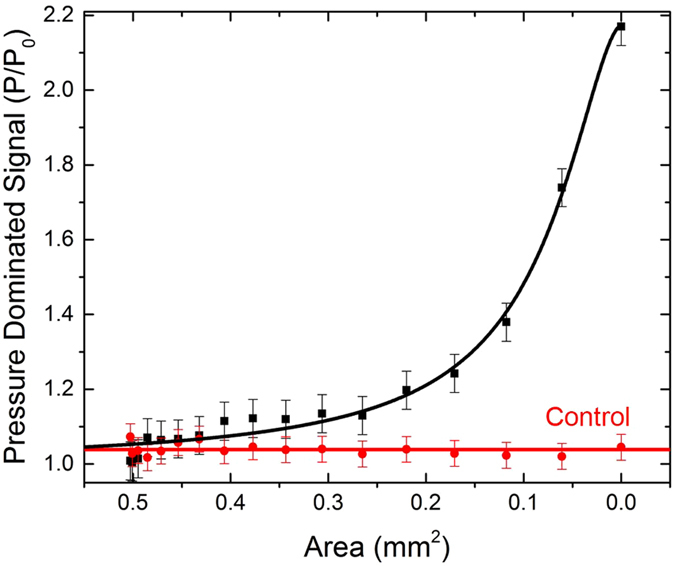
Distal occlusion results. Data showing pressure-dominated signal as a function of area for a distal occlusion. The signal has in it a small correction due to decreasing flow. As cross-sectional area decreases, it is possible to forecast when an occlusion is forming at the distal end of the shunt.

**Figure 4:**
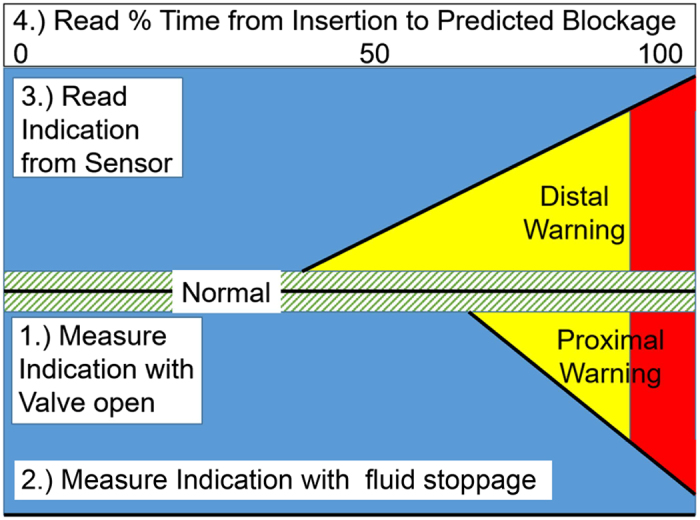
A method for forecasting shunt occlusions. Schematic graph of the method to assess the effects of occlusions or treatments on the flow and pressure of the cerebrospinal fluid in a smart shunt system. The picture is based on the data in Figs 2 and 3.

The method can be summarized as follows using Fig. 4:

1. First, the normal behavior may be measured with the pressure control valve set to a standard pressure at which it would be open as much as possible. The normal condition is given by the crosshatched band at a constant value.
2. Second, a check on a calibration of the sensor could be made by preventing flow through the system. In this case, a stoppage upstream of the sensor is equivalent to a blockage such as in a ventricular cavity. When the sensor calibration is a constant, the control measurement is a straight line.
3. When a ventricular occlusion becomes significant, the indication begins to decrease, as shown by the yellow area and when the occlusion nears complete blockage, the indication is red, following the data in Fig. 2. When a peritoneal (distal) occlusion occurs, the data in Fig. 3 show an indication of the opposite sign following a similar growth.
4. The estimated time to blockage can be estimated from the time-axis of the figure, which shows the % of the time since implantation. For instance, if the danger of a proximal occlusion is indicated in the dangerous (red) region for a shunt that was implanted about a year ago, the time to blockage is about 20% of full blockage or over a month. At this point, there is time for confirmation, further tests, and scheduling a shunt revision before serious brain damage occurs.

Tests show that this method presents the promise of early warning of blockage in shunt systems. Such a device has been sought since the invention of the shunt in 1956 because shunts are needed by patients for periods of decades and occlusions frequently form. An implanted shunt with an effective sensor opens up new fields of information about brain illnesses, such as idiopathic normal pressure hydrocephalus.

## Methods

**Figure 5:**
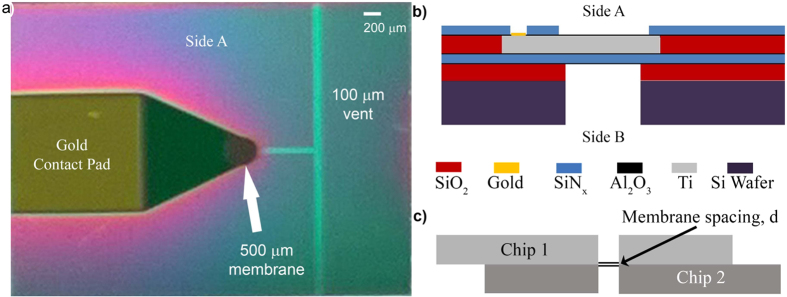
Smart shunt sensor. (**a**) Image of Side A of half a sensor. A complete sensor consists of two chips sandwiched together to construct a capacitor with Side A of both chips touching each other. The main feature is the 500 μm circular membrane that is attached to a 2 mm x 2 mm gold contact pad. Also visible is a 100 μm wide channel to act as a vent for air when chips are sandwiched together. Side B of the sensor is not shown, but has a through hole, which allows the fluid to make contact with the backside of the sensor. (**b**) Schematic demonstrating the cross sectional view and the various materials utilized making a smart shunt sensor. All materials for the sensor are biocompatible. (**c**) Not-to-scale cartoon schematic of a cross-sectional view through the hole of a complete sensor composed of two chips. As pressure gets exerted on the membranes, the membrane spacing decreases. Side B of both chips makes contact with the fluid environment, while Side A of each chips makes contact with one another.

The smart shunt studied here addresses the challenges of monitoring CSF using conventional principles of hydrodynamics, and uses state-of-the-art MEMS fabrication technology and electronics. A cross-sectional schematic of the chip and its materials can be seen in Fig. 5b, and a more detailed process flow can be seen in a previous publication^1^. The main features of the sensor are two circular flexible membranes 500 μm in diameter with a nominal spacing of <1 μm forming a capacitor (Fig. 5). The double-membrane sensor reported in this paper is formed by sandwiching two chips with flexible membranes together. Said sensor is housed in a package that allows compatibility with a fluid environment. Chen, *et al*.^26^ previously reported a capacitive pressure sensor capable of detecting pressures as low as 3 Pa. Utilizing the method presented in the Discussion section, any similarly well-designed sensor may be utilized as long as it is compatible with a fluid environment.

The capacitive sensor forms part of a resonant circuit in which an increase in the fluid pressure next to the flexible membrane decreases the distance between the membranes and increases the capacitance, *C*, in the circuit which also contains an inductor, *L*. An external reader couples inductively through the skin to the implanted circuit and measures its resonant frequency, 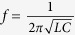
27. Utilizing a custom LabVIEW program to perform a high-speed averaging technique, a highly repeatable reading of the resonant frequency is achieved. The high repeatability of the frequency measurement allows the user to calculate the capacitance, and in turn, determine the small changes in the distance between the membranes via the equation:


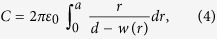


where *r* is the radius of the circular membrane, and *w(r*) is the membrane center deflection.

In careful studies carried out on wireless capacitive pressure sensors by Chen, *et al*.^26^, there was emphasis on the electronic loss in the environment of human tissue. By design, this sensor is to be sealed in a package to couple to the fluid flow and then encapsulated with the standard packaging used for variable pressure shunt control valves. No statistically significant shift between this *in vitro* simulation under artificial skin and a control measurement in air was observed.

**Figure 6:**
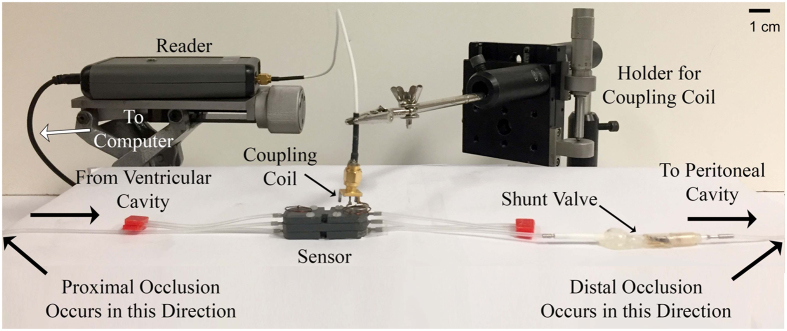
Experimental apparatus of simulated CSF flow path. The syringe pump (not shown) simulates the ventricles of the brain and standard shunt tubing is used as it flows into the smart shunt. Fluid drains into the artificial peritoneal cavity simulated by a Petri dish (not shown). Points at which occlusions occur are indicated in both directions.

The experimental set up is shown in Fig. 6. The fluid flows from the ventricular cavity (simulated by the syringe) into a standard sized shunt (0.8 mm internal diameter). Wells within the packaging house the capacitors out of the way of the flow. Rather than obstructing the flow, the wells ease the flow by increasing the diameter of the flow passage. The pressure differences at points along a calibrated length of the shunt tube allow a calculation of the flow if pressure remains constant. The fluid flows out of the system into the peritoneal cavity (simulated by a Petri dish).

Physiologically, flow of CSF is driven by the pressure gradient between the ventricular cavity and the blood brain barrier^28^ as fluid crosses during the production of CSF in the ventricles. In a patient with a shunt implant, the fluid flows through the tubing and into the peritoneal cavity where a lower pressure exists. In the reported measurements, a syringe pump with computer control of the flow rate is used to simulate the ventricular cavity and its production of CSF. The output flows into a conventional shunt tube. The fluid flows past one side of a double membrane sensor and is returned symmetrically to the other side. It is then drained to a digital scale at atmospheric pressure to simulate the peritoneal cavity^29^.

Figure 6 Also demonstrates how the sensor is read. The sensor is designed such that when a reading is taken, the user touches the surface of the skin with the reader’s coupling coil. As long as the reader is in place over the location of the sensor, the signal is very stable. If the user’s hand moves while holding the reader, it is possible that the signal could change. If the user is not touching the skin, but is positioned directly above the sensor, the reading will provide an accurate indication of the resonant frequency of the sensor, but the quality factor (Q-factor) of the signal will decrease. A substantial decrease in this signal makes it more difficult to distinguish the resonant peak from the rest of the signal. As long as the user touches their skin directly above the sensor, this issue does not arise. Another way the signal is effected is if the sensor’s coil and the reader’s coil are not aligned. In this case, the signal varies in Q and can vary in resonant frequency. Once the coils are misaligned enough, no signal is acquired. The design of the sensor is such that as long as the reader touches the skin at a stable point, the signal is quiet and only slight differences are present from one device to another (i.e. base capacitance). As long as the schematic presented in Fig. 4 is followed, it does not matter what the baseline reading is, since the changes in the signal are the data of interest.

All experiments were performed at room temperature with deionized water flowing through the system. In order to minimize the effect of stray electrical signals, the reader is calibrated prior to acquiring data by removing the sensor from the proximity of the reader and running a custom LabVIEW program. In addition, to minimize the effect of any vibrations present in the system, a high-speed averaging technique is utilized. This technique is expanded upon in the Statistics section. These two methods help to minimize stray signals. However, any stray signal in the 150 to 350 MHz range could potentially interfere with the reading once it is implanted.

### Statistics

The cerebrospinal fluid in the brain has dynamics associated with the blood brain barrier with slow, typical flow rates of 0.35 mL/min, or about 21 mL/hr^10^,^28^. Multiple reports of ICP have been given spanning ranges of 2 to 16 mmHg^22^ and 5 to 15 mmHg^20^. In one report, it spans as high as 18 mmHg^21^. In order to detect the hydrodynamics of the fluid, devices with significant sensitivity are needed. In designing our capacitive sensors, we estimated that a sensitivity of approximately 20 Å in the motion of the membrane would be needed to indicate that there was blockage or not. This corresponds to changes in pressure of about 0.13 mmHg (17 Pa).

The sensor detects pressure and derives flow from a pressure difference through a path that imparts a resistance to the flow. Before an occlusion upstream from the sensor, the maximum pressure will be the intracranial pressure. When the path is fully occluded, the minimum pressure will be that of the peritoneum, which for our experiments was set to the atmospheric pressure. Assuming that 5 mmHg is normal for a patient (below 10 mmHg is considered clearly normal^30^), then the sensor will detect the pressure drop from this to 0 mmHg when the shunt is proximally occluded. This is a more stringent case due to the steeper decline in signal. If we arbitrarily set a 10% decline as the requirement for early detection, then the sensor must be able to detect a change of 0.5 mmHg. We further impose that the variation in the signal be such that the 95% confidence limit separates the measurements of Δ*P* of 0.5 mmHg. That is, 1.96*σ* = 0.5 Δ*P* or 0.25 mmHg. This gives a required 
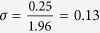
 mmHg or 17 Pa. The reported sensor is 17 times more sensitive.

The resonant circuit reader is coupled to inductors in the sensor circuits wirelessly, allowing measurements to be made through artificial skin simulating an implanted sensor. The position of the reader is fixed and a resonant sweep of 1,000 data points is acquired. In order to minimize stray vibration signals, a sample size of 15–30 trials per physiological parameter were performed, allowing over 15,000 data points per parameter. From this amount of data, the peak of the averaged values is determined through a custom Python code. Therefore, the results demonstrate the average of these many points. Over an entire experiment, as many as 270,000 data points were acquired, taking 2.5 to 5 minutes to run. This high-speed averaging enhances the sensitivity of the device.

All data presented passed the chi-squared test for normality. Statistics were performed on the data presented in Fig. 1 with standard deviations given above. The p-value for the control experiment was 0.0942, suggesting that the null hypothesis of no measurable shift in signal is upheld. For variable flow, it was less than 0.0001.

In Fig. 2, a p-value of 0.81 exists, suggesting that the null hypothesis of no measurable effect on the signal due to the changes in flow and pressure is upheld for the control. The p-value from no occlusion to an area reduced by 70% is 0.9999 showing that the null hypothesis of no measurable change in signal is appears to be upheld with a 99.99% probability over that range. For the formation of proximal occlusion data, the p-value is less than 0.0001, which suggests that the null hypothesis is violated. The p-value is so small because it is a comparison with the average value of the signal. This occurs when considering the region beyond 70% occlusion and we predict a measurable shift in signal in this range.

In Fig. 3, the case of distal occlusion formation, the p-value for the control is once again 0.81. However, in a region from no occlusion to an area reduced by 20% it is 0.9999 suggesting that the null hypothesis is upheld. Beyond that, the p-value is less than 0.0001, which leads to the conclusion that the null hypothesis of no measurable shift in signal is violated when a distal occlusion is forming. As in the case of the proximal occlusion, the p-value is small because it is a comparison with the average value of the signal. Further discussion on the statistics for simulated occlusions follow.

The occlusion study was performed by systematically changing the cross-sectional area of the shunt tubing. Digital calipers were used to alter the shape of the circular tubing to that of an ellipse with smaller cross-sectional area over a range of 0.5 mm^2^ (unobstructed) to 0 mm^2^ (completely occluded). The experiment was performed simulating proximal and distal occlusions.

Data in Fig. 2 demonstrates a logistic fit. In Fig. 2, the flow rate, *V*, decreased consistently with the function:


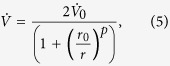


where, 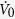 is the initial value of 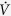 and *r*_0_ is the initial radius of the tube. The analysis shows the best fit for *p* = 4.02 ± 0.1, consistent with the expectation for lamellar behavior where the flow resistance varies as, 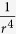. The agreement of the expected and observed exponent suggests that the behavior is determined primarily by flow in the balanced, calibrated flow path, with indication that flow dominates the pressure differences that occur in the entire shunt along with occlusion formation.

The functional form for Fig. 3 is given by a logistic fit:


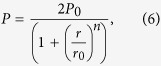


where, *P*_0_ is the initial value of *P* and *r*_0_ is the initial radius of the tube. When the occlusion is complete, *r* goes to 0 and *V* = 0. The analysis shows the best fit for *n* = 1.67 ± 0.1, which is not a rapid as 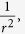
or dominated by pressure, but is probably also affected by the change in flow rate which is of the opposite sign. It is therefore considered consistent with the expectation for lamellar flow dominated by pressure as it rises approximately as the occluded area, 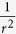. That is, the behavior is primarily a pressure increase between the occlusion and the ventricles.

Figure 2 Suggests that the pressure saturates; however, as long as the ICP continues to increase, the pressure-dominated signal will continue to increase. This holds true until a point where the membranes can no longer flex, but is unlikely to occur at the pressure levels being monitored. The data in Fig. 3 represents constant pressure. This explains why we see the data appearing to saturate. If the pressure remains constant, it will level off as demonstrated by the data; however, if it varies, the trend will continue.

Supplementary Figure 1 Demonstrates the statistics for Figs 2 and 3. Standard deviations for the variation in flow/pressure-dominated signal indicate the sensitivity of the signal when an occlusion is forming. In the case, when no occlusion is present, the standard deviation is 0.013. Once an occlusion is forming, standard deviation is 0.039 (proximal) and 0.026 (distal).

In order to further test repeatability of the results, multiple experiments were run over the course of one month. Three *in vitro* tests were performed in the proximal case, while four were performed for the distal case. The same trends were observed for both proximal and distal occlusions with minor changes due to fluctuations in atmospheric pressure. Since the peritoneal end is kept at the atmospheric pressure of the room, starting signal shifts were observable. This can be summarized as observing a different starting frequency before experimentation of 1 to 4 MHz depending on atmospheric pressure.

## Conclusions

The tests presented here demonstrate that it is possible to utilize an ultra-sensitive MEMS capacitor for monitoring the dynamics of CSF in shunt systems. Not only is the sensor able to monitor occlusions in shunt systems non-invasively, it can also predict when occlusions are forming months in advance. These predictions can aid in limiting the amount of brain damage patients with occluded shunts experience and improve their quality of life. This is a significant advance in shunt technology.

## Additional Information

**How to cite this article:** Apigo, D. J. *et al*. Evidence of an application of a variable MEMS capacitive sensor for detecting shunt occlusions. *Sci. Rep.* **7**, 46039; doi: 10.1038/srep46039 (2017).

**Publisher's note:** Springer Nature remains neutral with regard to jurisdictional claims in published maps and institutional affiliations.

## Supplementary Material

Supplementary Information
