# Supplementary material for: Evidence of an application of a variable MEMS capacitive sensor for detecting shunt occlusions
Source: Sci Rep. 2017 Apr 5;7:46039. doi: 10.1038/srep46039 (PMC5380964; doi:10.1038/srep46039)
Supplement: Supplementary Information [file srep46039-s1.pdf]

Evidence of an application of a variable MEMS capacitive sensor for detecting shunt occlusions: Supplementary Figures

by
David J. Apigo, Philip L. Bartholomew, Thomas Russell, Alokik Kanwal, Reginald C. Farrow, Gordon A. Thomas

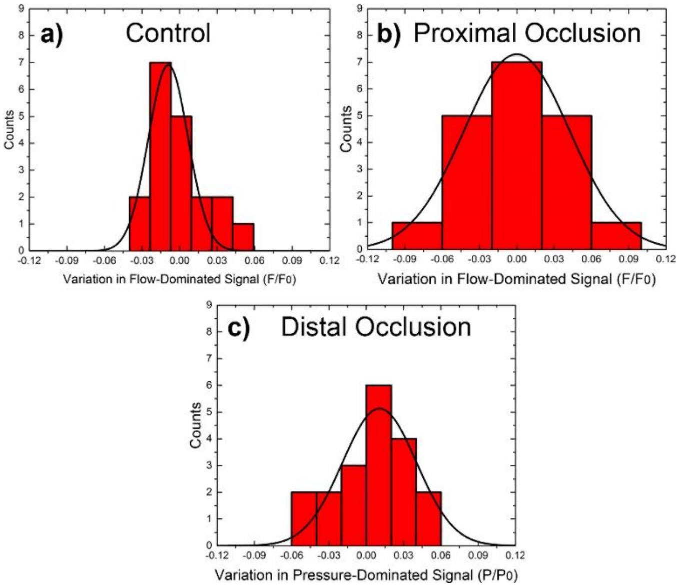

Supplementary Figure 1. Statistics for Occlusion Results. Histograms demonstrating standard deviation for control standard deviation, $\sigma = 0.013$, b) proximal catheter occlusion, $\sigma = 0.039$ and c) distal catheter occlusion, $\sigma = 0.026$.
